# Physicochemical Nature of SARS-CoV-2 Spike Protein Binding to Human Vimentin

**DOI:** 10.1021/acsami.3c03347

**Published:** 2023-07-06

**Authors:** Piotr Deptuła, Krzysztof Fiedoruk, Monika Wasilewska, Łukasz Suprewicz, Mateusz Cieśluk, Paulina Żeliszewska, Magdalena Oćwieja, Zbigniew Adamczyk, Katarzyna Pogoda, Robert Bucki

**Affiliations:** † Independent Laboratory of Nanomedicine, Medical University of Bialystok, PL-15222 Białystok, Poland; ‡ Department of Medical Microbiology and Nanobiomedical Engineering, Medical University of Bialystok, PL-15222 Białystok, Poland; § J. Haber Institute of Catalysis and Surface Chemistry Polish Academy of Science, Niezapominajek 8, PL-30239 Krakow, Poland; ∥ Institute of Nuclear Physics Polish Academy of Sciences, PL-31342 Krakow, Poland

**Keywords:** SARS-CoV-2, vimentin, single-molecule binding interactions, single-molecule force spectroscopy, quartz crystal microbalance

## Abstract

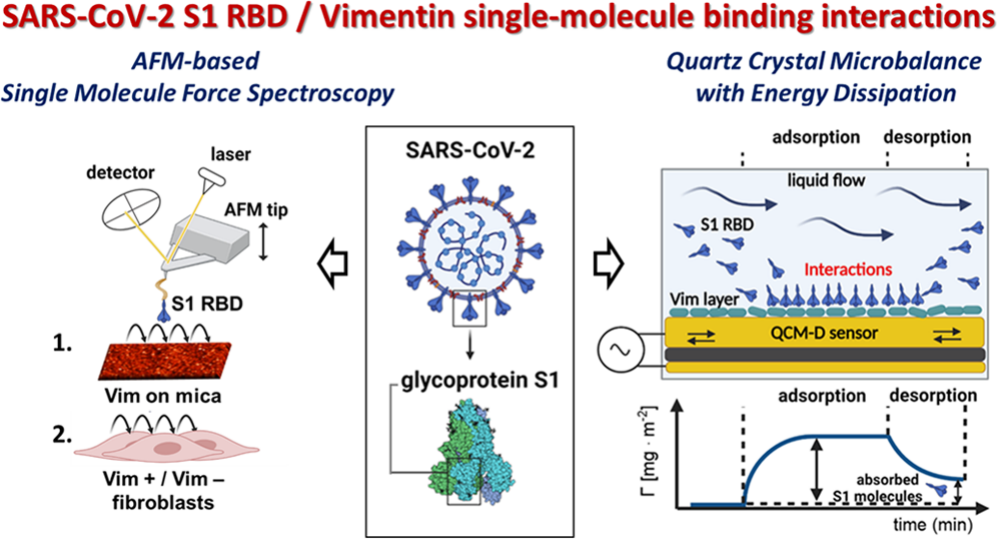

Vimentin, a protein that builds part of the cytoskeleton and is involved in many aspects of cellular function, was recently identified as a cell surface attachment site for the severe acute respiratory syndrome coronavirus 2 (SARS-CoV-2). The present study investigated the physicochemical nature of the binding between the SARS-CoV-2 S1 glycoprotein receptor binding domain (S1 RBD) and human vimentin using atomic force microscopy and a quartz crystal microbalance. The molecular interactions of S1 RBD and vimentin proteins were quantified using vimentin monolayers attached to the cleaved mica or a gold microbalance sensor as well as in its native extracellular form present on the live cell surface. The presence of specific interactions between vimentin and S1 RBD was also confirmed using in silico studies. This work provides new evidence that cell-surface vimentin (CSV) functions as a site for SARS-CoV-2 virus attachment and is involved in the pathogenesis of Covid-19, providing a potential target for therapeutic countermeasures.

## 1 Introduction

The S-protein is the major viral attachment protein (VAP) of severe acute respiratory syndrome coronavirus 2 (SARS-CoV-2) that interacts with the human angiotensin-converting enzyme 2 (ACE2) receptor, which is the key intermediary in the entry of the virus.^1−3^ Recent studies indicate that the interaction of viral proteins with ACE2 alone may not be sufficient to ensure optimal entry of the virus into cells, and the process is enhanced by other factors.^3−5^ Several potential SARS-CoV-2 receptors/coreceptors, such as integrins, heparan sulfate, sialic acid, AXL receptor tyrosine kinase, neuropilin-1, CD209L/L-SIGN, and CD209/DC-SIGN, have been described.^1,4,5^ In 2016, Yu et al.^6^ identified vimentin (Vim) as a critical factor for infection of cells by SARS-CoV-1. In addition, Vim has been recognized as the receptor or coreceptor for several other viruses.^7−9^ Vim exists in nonfilamentous extracellular forms, i.e., when attached to the cell surface or secreted into the extracellular space.^10^ We recently observed that anti-Vim antibodies significantly block SARS-CoV-2 pseudovirus entry into ACE2-expressing cells.^11^ Vim was also identified as an attachment factor supporting SARS-CoV-2 entry into human endothelial cells.^12^

In this study, the intermolecular forces between the SARS-CoV-2 S1 RBD and human Vim layers were quantified using atomic force microscopy (AFM)-based single-molecule force spectroscopy (AFM-SMFS) and a quartz crystal microbalance with energy dissipation (QCM-D). Additionally, the AFM technique was employed to study not only the wild-type S1 RBD–Vim complex formation but also different SARS-CoV-2 variants of concern (VOC)—Brazil, South Africa, and United Kingdom. Mouse embryonic fibroblasts expressing Vim (mEF +/+) and their Vim null counterparts (mEF −/−) served as the cellular system to study the S1 RBD and native extracellular Vim interactions using AFM force spectroscopy measurements. Experimental results were followed up by molecular dynamics simulations of Vim and S1 RBD docking prediction. Our studies can serve as the multimodal proof for specific interactions between extracellular Vim and S1 RBD proteins engaged in SARS-CoV-2 virus entry and provide the very first physicochemical characterization of this interaction.

## 2 Experimental Section

### 2.1 Materials and Methods

In our study, we used single-molecule force spectroscopy–atomic force microscopy to evaluate the binding probability and strength of the interaction between the S1 RBD and Vim. To study the interaction between the S1 RBD and Vim, the AFM probe was functionalized using a poly(ethylene glycol) (PEG) linker. The specific interactions between the wild-type strain S1 RBD and Vim were also confirmed using a quartz microbalance. This technique was used for precise, real-time measurement of Vim/S1 RBD bilayer adsorption and desorption kinetics under flow conditions. In the first experimental step, interactions between the S1 RBD and Vim on a mica surface were investigated. Once the specificity of S1 RBD and Vim molecules was confirmed using AFM-SMFS and QCM-D techniques, interaction forces between the S1 RBD and CSV on mouse embryonic fibroblasts (mEF +/+ and mEF −/−) in physiological conditions were investigated. For the cellular model, interactions between Vim and the S1 RBD from selected VOCs—Brazilian, South African, and the U.K.—were also investigated.

#### 2.1.1 Cell Culture

Wild-type mouse embryonic fibroblasts (mEF +/+) and their Vim KO variant (mEF −/−) were grown in Dulbecco’s modified Eagle’s medium (ATCC, #30-2002), supplemented with 10% fetal bovine serum (PAN Biotech, #P30-8500) and 1% antibiotic antimycotic solution (Sigma-Aldrich, #A5955) at 37 °C in a humidified atmosphere with 5% CO_2_. For AFM experiments, 10^5^ of the cells were seeded onto a 35 mm plate (TPP, #93040) and left for 5 days in an incubator until a fully confluent monolayer was formed.

#### 2.1.2 SARS-CoV-2 Spike RBD Proteins

Recombinant SARS-CoV-2 receptor binding domain spike proteins (S1 RBD) derived from several strains of the coronavirus were used for AFM tip functionalization. Namely, the wild-strain SARS-CoV-2 spike protein RBD (RayBiotech, #230-01102-1000) and three variants of concern (VOC) were purchased from SinoBiological, Brazil P.1 RBD (#40592-V08H86), South Africa B.1.135 RBD (#40592-V08H85), and United Kingdom B.1.1.7 RBD (#40592-V08H82). Recombinant spike RBD proteins were aliquoted at 0.1 mg/mL in phosphate-buffered saline (PBS) (Gibco, #10010-015).

#### 2.1.3 Fluorescent Staining and Imaging

Vital staining was performed to visualize extracellular Vim. First, an anti-Vim antibody (Abcam, #ab92547) at 1:500 dilution was introduced to a confluent cell monolayer grown on a glass coverslip for 1 h at 4 °C. Cell fixation was performed with the use of a 3.7% solution of paraformaldehyde in PBS for 20 min at room temperature (RT). Subsequently, in order to block nonspecific binding, 0.1% of bovine serum albumin (BSA) in PBS was added for 30 min at RT. A secondary antirabbit Alexa Fluor 488 antibody (Abcam, #ab150081) was used at 1:1000 dilution for 1 h at RT in the dark. Nuclei were counterstained with NucBlue Live ReadyProbes Reagent (ThermoFisher, #R37605) according to a protocol provided by the manufacturer. In a parallel set of experiments, staining of intracellular Vim was performed. In contrast to vital staining, after the fixation step, cells were permeabilized with 0.1% Triton X-100 (Sigma-Aldrich, #X-100) for 15 min at RT prior to staining. Each step was followed by extensive PBS washing. Extracellular and intracellular Vim were visualized with the use of a confocal microscope.

#### 2.1.4 Preparation of Vim-Coated Mica Surfaces

Vim was immobilized onto mica surfaces coated with poly-l-lysine. The day preceding measurements, 100 μL of 0.01% poly-l-lysine solution (Sigma-Aldrich, #25988-63-0) was incubated with freshly cut mica slices. The next day, 10 μL of 0.1 mg/mL Vim solution in PBS was dropped onto the dried mica surface and rinsed with PBS after 30 min of incubation at room temperature.

#### 2.1.5 AFM Measurements

**Figure 1:**
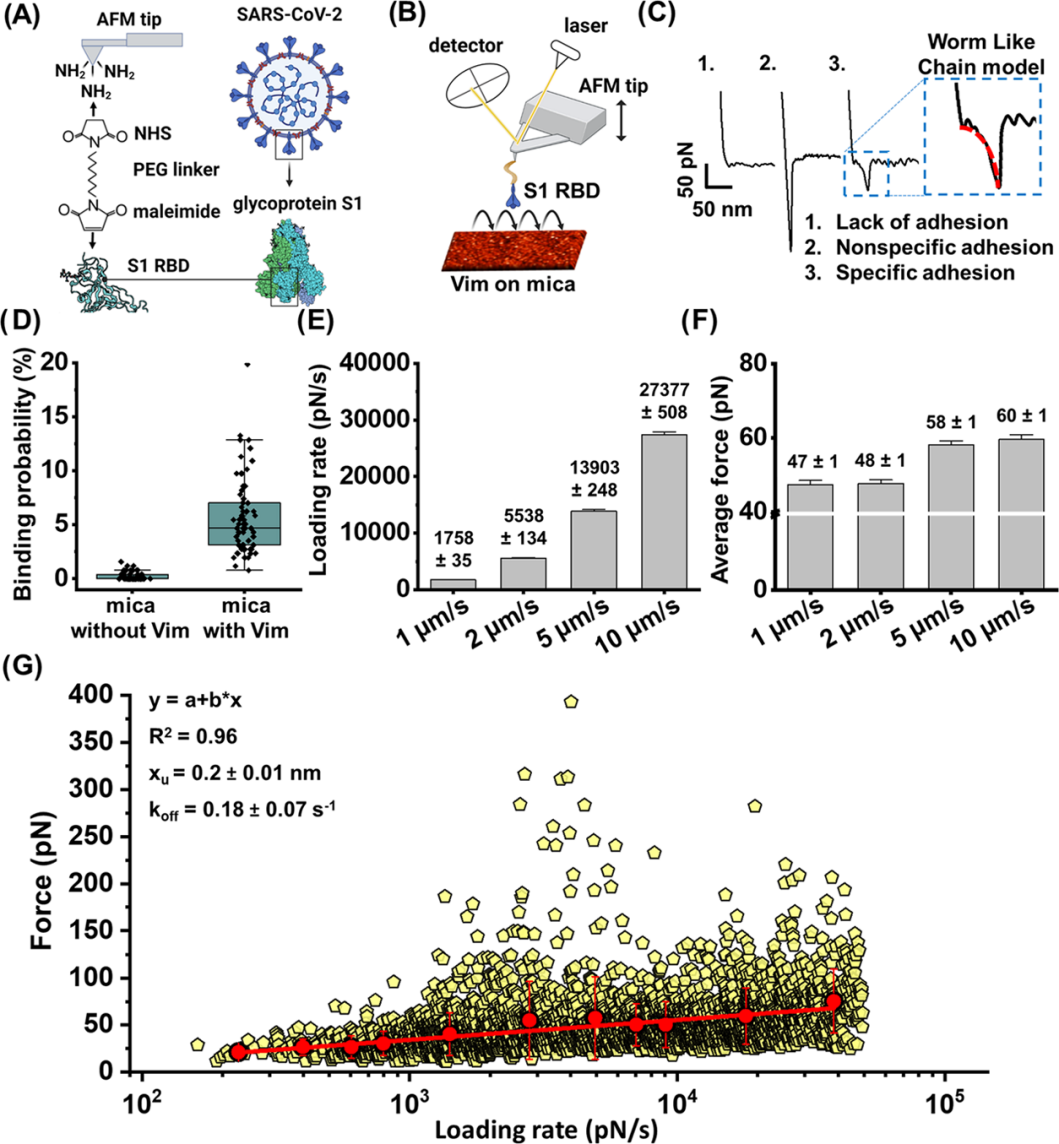
Measurement of S1 RBD and Vim interactions using AFM. (A) Schematic representation of the process of AFM tip functionalization with the S1 RBD subunit protein. (B) Silicon nitride AFM cantilevers were used to probe the interactions between wild-strain S1 RBD and Vim immobilized on mica. (C) Analysis of force–distance (FD) curves to determine the reaction forces between the S1 RBD and Vim. The retraction part of FD curves was used for curve classification. Panel (C1) shows a nonadhesive curve collected from the mica surface without Vim. Panel (C2) shows nonspecific adhesion occurred between the S1 RBD and mica surface. Such curves were not considered for analysis. Panel (C3) shows a specific adhesion event between the S1 RBD and Vim on the mica surface and such curves were taken into account for further calculations. (D) Box plot of specific binding probabilities measured by AFM between wild-strain S1 RBD and mica with immobilized Vim, as well as control mica surfaces with poly-l-lysine and no Vim. One data point belongs to the one force map and denotes the percentage of adhesive force curves in relation to all force curves acquired in this map. (E, F) Reaction forces and corresponding loading rates detected for different AFM probe retraction speeds (1, 2, 5, and 10 μm/s). (G) Distribution of the rupture forces as a function of their natural logarithm of loading rates (log LR) measured between the wild-strain S1 RBD and Vim on mica. One yellow point corresponds to one force–distance curve with the worm-like chain model fitted using JPK data processing software that allowed for loading rate determination (*N* = 2843, yellow data points). In order to describe the S1 RBD–Vim bond as a two-state model (bound and unbound states), the Bell–Evans prediction of linear dependence of the rupture force on the log of loading rates was used.^4,23^ The solid line on panel (G) represents the linear fit (*y* = *a* + *bx*) of all mean data points with subsequent calculation of the single energy barrier width and dissociation rate with the Bell–Evans model.^4,24,25^ For every LR range, mean values of the force between the wild-strain S1 RBD and Vim were determined (red dots). The error bars indicate the standard deviation (SD) of every mean force value for dataset in the loading rate ranges. Using the slope of the fit, the width of the energy barrier (*x*_u_) was estimated, while the intercept of the fit was used to calculate kinetic off-rate *k*_off_ (dissociation rate). For measurement of the S1 RBD and Vim interactions and distribution of the rupture forces, the reaction forces occurring in the LR range up to 50000 pN/s were considered. Experiments were repeated 3–4 times with independent tips and sample preparation.

AFM was used to evaluate the single-molecule binding interactions between the S1 RBD and Vim on the mica surface and cell models (Figure 1). The interaction was measured using a NanoWizard 4 BioScience JPK Instruments Bruker atomic force microscope working in the force spectroscopy mode. To investigate the S1 RBD–Vim interactions, the AFM force–distance (FD) curves were recorded. AFM measurements of Vim immobilized on the mica surface were performed in PBS at room temperature. Vim +/+ and −/– fibroblasts were measured on a Petri dish heater at physiological temperature (37 ± 1 °C). The interactions between the S1 RBD and Vim on living cells were investigated in a filtrated DMEM with 10% bovine serum. To rule out the potential interference of S1 RBD with ACE2 binding in our cellular model, we have used cells expressing surface Vim but no ACE2 receptors for the experiments. Force–distance curves were collected from different places on the sample (fresh mica slices with immobilized Vim or Petri dish with confluent fibroblasts). Up to 20 force maps consisting of 16 × 16 points corresponding to a scan area of 5 μm × 5 μm were acquired for each sample, taken from multiple random places on mica or confluent cell surfaces. For all measurements, 0.15 nN of the set point was used. 1, 2, 5, and 10 μm/s AFM cantilever approach/retraction speeds were used for probing recombinant human Vim immobilized on mica. The wild-strain S1 RBD-mEF +/+ interactions were probed at 1, 5, and 10 μm/s approach/retraction speeds, while South African, Brazilian, and U.K. variants were tested at a single speed of 5 μm/s; therefore, the lower number of data points are recorded for the VOC-derived S1 RBD. For the analysis of specific adhesion events, a worm-like chain model providing reaction forces and loading rate (LR) data (JPK data processing—JPK build-in analysis software) was used. The retraction part of obtained curves was used for curve classification and the S1 RBD and Vim interaction determination, as presented in Figure 1C3. Curves with specific adhesion show a very characteristic “shape” that can be fitted using the worm-like chain (WLC) model. JPK software was used for the WLC model fitting in the part of the force curve denoting the stretching of the polymer and the corresponding loading rate (LR) values were simultaneously determined. Results of the mean forces and LR are the average from all collected force–distance curves in which the adhesion forces were observed. OriginPro was used to display the average force and loading rate results and for all distributions of the rupture forces as a function of their LR.

#### 2.1.6 Functionalization of AFM Tips

Silicon nitride AFM cantilevers (Bruker, MSCT-C) with a nominal spring constant of 0.01 N/m were used to probe the interaction between SARS-CoV-2 S1 RBD proteins and recombinant human Vim (SinoBiological, 10028-H08B) immobilized on mica. In another set of experiments, interactions were measured between the S1 RBD and Vim presented in its native form on the cell surface. SARS-CoV-2 RBD proteins were attached to AFM cantilevers by means of a maleimidopropionyl–PEG–NHS heterobifunctional linker (Sigma-Aldrich, #689777) as previously described.^4,13^ AFM tips were aminosilanized, and a PEG linker was anchored to the amino group bearing tips through its NHS end. S1 RBD was attached to the PEG linker with its free end via a maleimide–cysteine bond. AFM tips were immersed in chloroform (POCh SA, #BA4431116) for 10 min, rinsed with ethanol, dried with a stream of nitrogen, and sterilized for 10 min using ultraviolet radiation. Tips were immersed overnight in an ethanolamine solution containing 3.3 g of ethanolamine hydrochloride (Sigma-Aldrich, #RDD028) in 6.6 mL of DMSO (POCh SA, #363550117). The next day, tips were washed thrice with DMSO and ethanol, respectively. Ethanolamine-treated cantilevers were immersed in maleimidopropionyl–PEG–NHS solution (3.3 mg of it was diluted in 0.5 mL of chloroform and 30 μL of triethylamine (Sigma-Aldrich, #T0886)) and then washed with chloroform 3 times and dried with nitrogen. At this point, the NH_2_ group on the AFM tip forms an amide bond with the NHS ester end of the linker. The subsequent step involved binding of the thiol group in the protein of interest with the maleimidopropionyl end of the linker;^14^ briefly, 50 μL drop of S1 RBD solution (0.1 mg/mL) was put onto the cantilevers placed on a parafilm (Bemi, #13-374-12) and 2 μL of fresh NaCNBH_3_ (Sigma-Aldrich, #156159) solution (6% wt vol^–1^ in 0.1 M NaOH(aq)) was mixed in the protein solution. The cantilevers were incubated in the solution for 1 h on ice. Then, 5 μL of 1 M ethanolamine solution (Sigma-Aldrich, #E9508) was carefully added to the protein solution and incubated for additional 10 min to quench the reaction and finally rinsed three times with PBS to wash off unbounded spike protein. Functionalized AFM cantilevers were kept in PBS and used in AFM experiments within the same day. This type of tip functionalization technique has been widely described in previous works.^4,15^

#### 2.1.7 Quartz Crystal Microbalance Measurements

The quartz crystal microbalance with dissipation monitoring (QCM-D) technique was used for precise, real-time investigations of Vim/S1 RBD bilayer adsorption and desorption kinetics under flow conditions. In these measurements, quartz sensors with the silicon dioxide (SiO_2_) layer, a commercial product of Q-Sense, Gothenburg, Sweden, were used. Before each experiment, the sensors were cleaned with a mixture of 95% sulfuric acid (H_2_SO_4_), hydrogen peroxide (30%), and deionized water in a volume ratio of 1:1:1 for 5 min. Then, they were rinsed with deionized water, heated in water at 80 °C for 30 min, and dried out in a stream of nitrogen gas. The roughness of sensors was examined by atomic force microscopy (AFM) imaging according to the procedure described in ref (16). It has been shown that the sensors were smooth, having the root mean square (rms) roughness below 1 nm (up to 15 AFM maps, each from an area equal to 4.0 μm^2^). Our method based on the desorption kinetics is advantageous compared to the classical method, where the amount of adsorbed molecules is determined and fitted by a Langmuir-type isotherm.^17^ Given the large adsorption constant for the second fraction approaching 10^9^ M^–1^, the latter method would require QCM-D measurements to be performed at a pM concentration range of S1 RBD that would require excessively long adsorption times prone to a large experimental error.

In our previous studies, we have already described adsorption kinetics and formation of Vim layers on solid substrates, including negatively charged mica and silica and polymer particle surface.^18^

#### 2.1.8 QCM-D Experimental Procedure

The experimental procedure was as follows: at the beginning of the measurement, a stable baseline was determined for the pure electrolyte of controlled ionic strength and pH. The volumetric flow rate of pure electrolyte was varied between 0.05 and 0.15 cm^3^ min^–1^. After the baseline stabilizes, the Vim solution of the concentration varied between 5 and 20 mg L^–1^ was flushed under a controlled flow rate. After obtaining a stable frequency and dissipation signal, the pure electrolyte solution was again flushed to remove weakly bound molecules. After depositing a Vim layer of a controlled coverage, in the next step, the S1 adsorption was carried out under the same ionic strength, pH, and flow rate for the protein solution concentration equal to 5 mg L^–1^. After obtaining a stable frequency shift signal, the desorption run was performed, where the pure electrolyte solution was flushed through the cell at the same flow rate. The S1 RBD desorption kinetics determined in this way was theoretically analyzed in terms of the general random sequential adsorption (RSA) model, which enabled to determine the Vim/S1 RBD binding energy quantitatively.^19^

#### 2.1.9 QCM-D’s Physical Fundamentals

**Figure 2:**
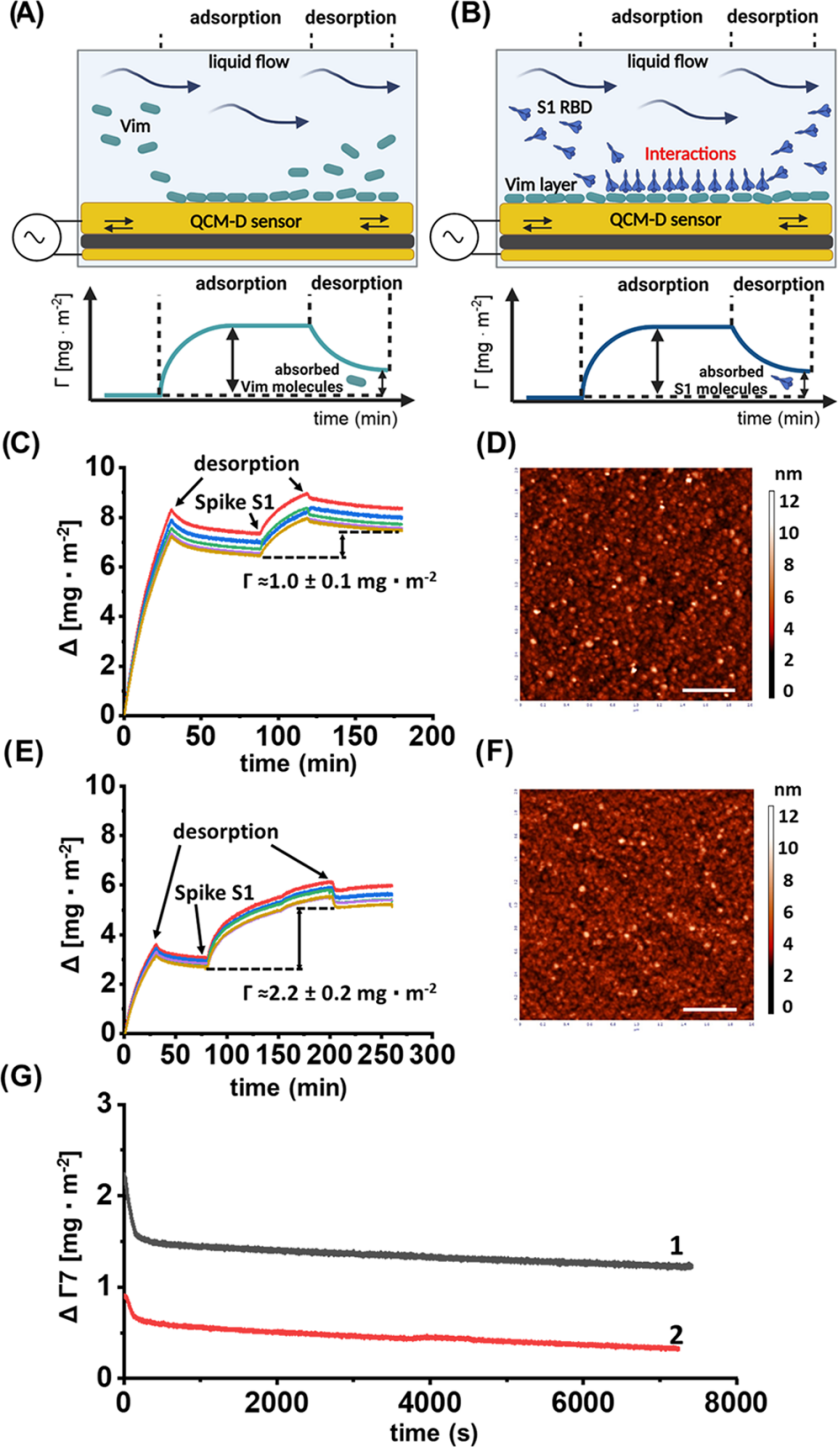
Kinetics of Vim/S1 RBD bilayer formation at the silicon oxide-coated sensor, monitored by QCM-D technology. (A, B) Schematic representation of measurements from the quartz crystal microbalance (overtones 3–11). A piezoelectric sensor is excited to resonance using an alternating voltage. The sensor’s resonance frequency, which depends on its mass (thickness), is monitored as a function of time. Frequency changes during time reveal changes in mass (mass uptake and mass loss) coupled to the sensor surface. These changes are used to analyze molecule–surface interactions and molecule adsorption and desorption. The experiment consisted of two parts. First, the adsorption of Vim molecules on the sensor surface and desorption of loose molecules were forced. Next, S1 RBD adsorption (by molecular interactions) and desorption of non-Vim-related molecules took place on the previously created Vim layer. (C–F) Vim adsorption/desorption kinetics is expressed as the coverage’s dependence (calculated using the Sauerbrey equation) on the deposition time; adsorption conditions: bulk protein concentration 5 mg L^–1^, flow rate 8.3 × 10^–4^ cm^3^ s^–1^. (C, D) pH 7.4, (E, F) pH 5.6. (D) AFM micrographs of protein monolayers at the silica sensor after the adsorption run for pH 7.4. Scale bar: 0.5 μm. (F) AFM micrographs of protein monolayers at the silica sensor after the adsorption run for pH 5.6. Scale bar: 0.5 μm. (G) Desorption kinetics of the S1 RBD investigated in situ by QCM-D, experimental conditions: ionic strength 0.15 M NaCl, pH 5.6, the flow rate of 0.05 cm^3^ min^–1^, line 1—bulk S1 RBD concentration equal to 20 mg L^–1^, line 2—bulk S1 RBD concentration equal to 5 mg L^–1^.

The adsorbed protein mass per unit area, hereafter referred to as the QCM-D mass (coverage) and denoted by Γ_Q_, was calculated from the Sauerbrey equation^20^
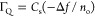
1where Δ*f* is the frequency shift depending on the overtone number *n*_o_ and is the Sauerbrey constant equal to 0.177 (mg^-2^ Hz^-1^) for the fundamental frequency *f*_F_ equal to 5 × 10^6^ Hz (*Z*_q_ is the acoustic impedance of the quartz sensor equal to 8.8 × 10^6^ kg m^–2^ s^–1^). The kinetics of the desorption runs shown in Figure 2 was interpreted in terms of the theoretical approach, where the following equation was used.^21^
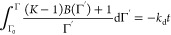
2where Γ_0_ is the initial coverage of the S1 RBD, *K* = *k*_a_/*k*_c_ is the dimensionless coupling constant, *B*(Γ′) is the blocking function derived from the RSA model, *k*_a_ is the kinetic adsorption constant, *k*_c_ is the mass transfer constant, and *k*_d_ is the kinetic desorption constant.

If *K* ≫ 1 and *B* ∼ 1, which is the usual case in protein desorption studies, eq 2 simplifies to the exponential form
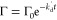
3where *k*_d_^′^ = *k*_c_/*K*_a_ and *K*_a_ is the equilibrium adsorption constant.

By fitting the experimental kinetic curves using numerical solutions of eq 1 or analytical solutions of eq 2, one obtains the desorption constant from which the equilibrium adsorption constant is calculated from the dependence
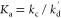
4The mass transfer rate constant was calculated from the dependence
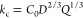
5where *C*_0_ is a known parameter depending on the QCM cell geometry (equal to 1.9 cm^-4/3^),^22^*D* is the S1 RBD diffusion coefficient acquired from DLS measurements, and *Q* is the flow rate of the suspension. It was calculated from eq 5 that *k*_c_ = 6.9 × 10^-5^ cm s^–1^ for the flow rate of 0.05 cm^3^ min^-1^.

The equilibrium adsorption constants were calculated from eq 4. The above *K*_a_ equilibrium adsorption constant is connected with the commonly used *K*_eq_ constant expressed in L mol^–1^^17^ via the linear dependence
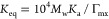
6where *M*_w_ is the molar mass and Γ_mx_ is the maximum coverage of the S1 RBD.

Therefore, the standard free energy of adsorption is given by
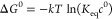
7where *c*^0^ is the reference concentration of the protein equal to 1 mol L^–1^.

## 3 Results and Discussion

In the current study, we have utilized AFM and QCM-D techniques to investigate the biophysical binding mechanism between the viral S1 RBD and Vim. The binding potential of S1 RBD by Vim was tested using mica as a model surface presenting Vim, as well as under cell culture settings with mouse embryonic fibroblasts characterized by the absence of the ACE2 receptor (mEF +/+), including the Vim KO variant (mEF −/−) as a control.

### 3.1 AFM-Based Single-Molecule Force Spectroscopy

In the first experimental step, we used single-molecule force spectroscopy–atomic force microscopy to evaluate the binding probability and strength of the interaction between the S1 RBD and Vim on a mica surface (see Figure 1 and Supporting Information). Figure 1 shows the S1 RBD–Vim interaction characterization on the model mica surface. Rigorous exclusion criteria for force curves analysis were applied with examples shown in Figure 1C. Figure 1D indicates a box plot of specific binding probabilities (BPs) with and without Vim molecules on top of the mica surface. Specific adhesion events for S1 RBD and a mica surface with immobilized Vim were observed on up to 20% of all registered force curves, while binding probability on mica without Vim was close to zero. Figure 1E,F shows mean values of loading rates (delivered from originally collected force–time AFM curves) and rupture forces detected for different AFM probe approach/retraction speeds of 1, 2, 5, and 10 μm/s. Increasing the AFM retraction speed increases the values of the detected forces and loading rates.

To test if S1 RBD–Vim and S1 RBD–ACE2 interactions occur at a similar force range, we have also immobilized ACE2 receptor on the mica surface. For an AFM approach/retract speed of 5 μm/s, the average rupture force between S1 RBD and ACE2 was 40 pN, which is lower than the S1 RBD–Vim rupture force of 58 pN when measured in a parallel experiment (Supporting Information, Figure S1). Panel (G) shows rupture force distribution as a function of their loading rates that were fitted to a single-barrier Bell–Evans model. The activation barrier width *x*_u_ for the S1 RBD and Vim immobilized on the mica surface was calculated to be 0.2 ± 0.01 nm, while the dissociation rate *k*_off_ was 0.18 ± 0.07 s^–1^ (at LR = 0).

### 3.2 Quartz Crystal Microbalance with Dissipation Monitoring

The specific interactions between wild-type strain S1 RBD and Vim were also confirmed using a quartz crystal microbalance with dissipation monitoring (QCM-D). Here, the interactions of the S1 RBD with preadsorbed Vim layers of controlled coverage were performed under flow conditions. A typical experiment performed in 0.15 M PBS, pH 7.4 and 0.15 M NaCl, pH 5.6, and a flow rate of 0.05 cm^3^ min^–1^ is shown in Figure 2. The amount of protein adsorbed depends on the pH of the solution. At pH 7.4, 1 ± 0.1 mg m^–2^ of S1 RBD was adsorbed, and at pH 5.6, 2.2 ± 0.2 mg m^–2^ was adsorbed. A thorough analysis of the kinetic curves presented in Figure 2 showed that there were two main fractions of the S1 protein: one loosely bound, characterized by a desorption time of 200 s, and the second more tightly bound with a desorption time exceeding 10,000 s. These fractions were characterized by the equilibrium adsorption constants equal to 0.0011 and 0.46 cm, respectively (see Table 1). The results shown in Table 1 confirm that there are two fractions of adsorbed S1 RBD characterized by free adsorption energies of −36 ± 1.2 kJ and −48 ± 1.7 kJ mol^–1^, respectively. The first fraction is probably formed at the top of one Vim molecule, whereas the second corresponds to the S1 molecules interacting with two or more adsorbed Vim molecules. The results clearly indicate specific interactions between S1 and Vim, confirming the AFM force spectroscopy results.

**Table 1:** Equilibrium Adsorption Constant and the Free Energy of Adsorption for the S1 RBD/Vim Interactions in Supporting Bilayers at a Silica Sensor Derived from QCM-D Measurements at *T* = 298 K

fraction	*k*_d_^′^ [s^–1^]	*K*_a_ [cm]	*K*_eq_ [L mol ^–1^]	Δ*G*^0^ [kT]	Δ*G*^0^ [kJ mol ^–1^]
I	0.063	1.1 × 10^–3^	4.7 × 10^6^	–15 ± 0.6	–36 ± 1.2
II	1.5 × 10^–4^	0.46	9.2 × 10^8^	–20 ± 0.6	–48 ± 1.7

**Figure 3:**
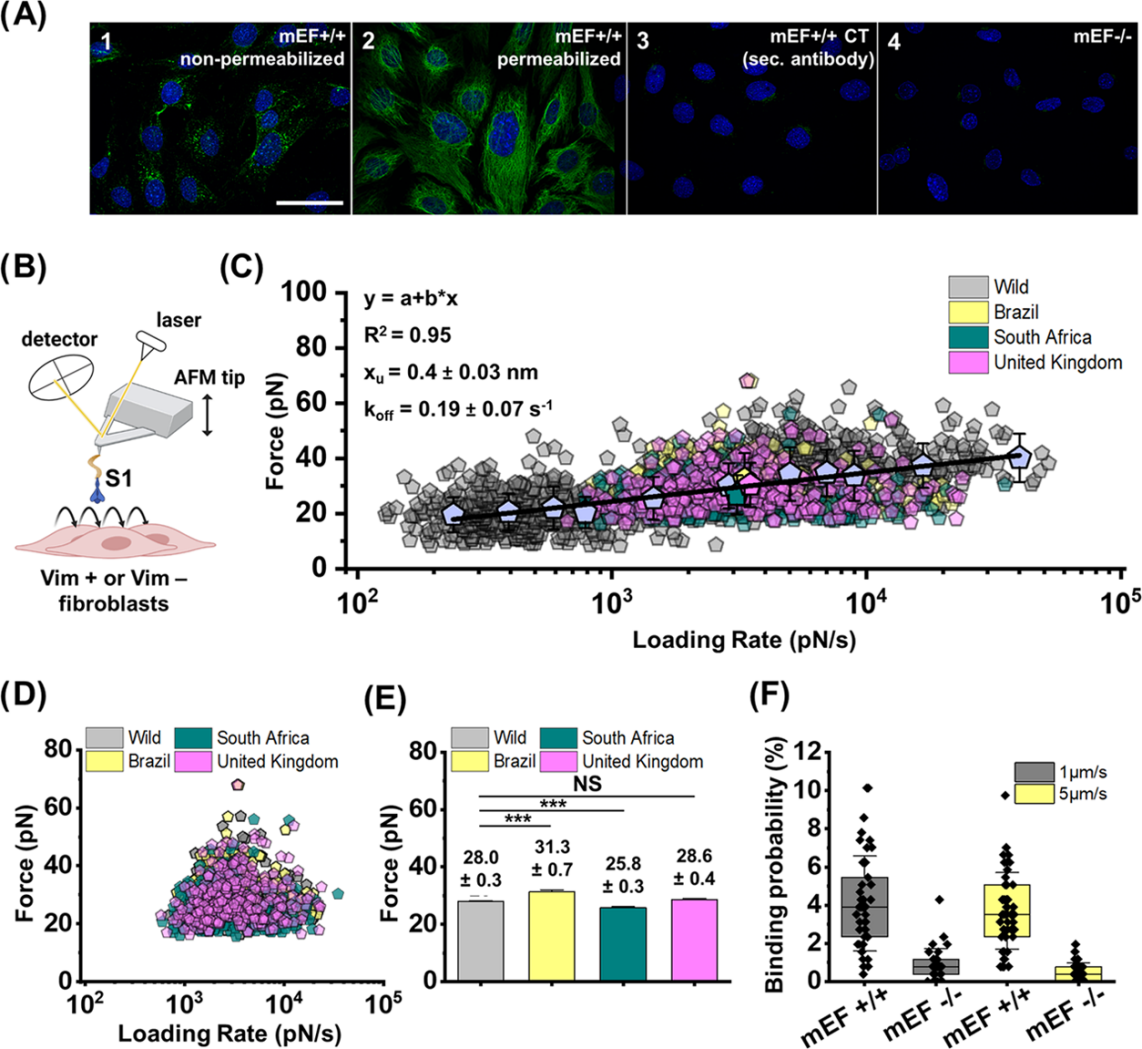
Probing S1 RBD interactions with CSV on living cells expressing Vim (mEF +/+) and their Vim null counterparts (mEF −/−). (A) Fluorescence images of Vim (green) visualized in immunostained mouse embryonic fibroblasts-expressing Vim mEF +/+ (A1–A3), and Vim KO mouse embryonic fibroblasts mEF −/– considered as a negative control (A4). Scale bar: 50 μm. (B) Idea of AFM-SFSM where S1 RBD is attached to the AFM probe and used in live cell experiments. (C) Distribution of rupture forces as a function of their loading rates (LRs) measured for the wild-strain S1 RBD (gray) and three S1 RBD VOCs—Brazil P.1 RBD (yellow), South Africa B.1.135 RBD (blue), United Kingdom B.1.1.7 RBD (purple), and live mEF +/+ cell surface [*N* = 1186 data points for wild strain (gray dots); *N* = 203 data points for Brazil (yellow dots); *N* = 297 for South Africa (blue dots); *N* = 308 data points for the United Kingdom (purple dots)]. For every LR range, mean values of the rupture force between the wild-strain S1 RBD and CSV were determined (big gray dots). The error bars indicate the standard deviation for every mean force value. The solid line is a linear fit of all data points for the wild-strain S1 RBD with the Bell–Evans model. (D) Comparison of rupture forces determined for all S1 RBD proteins and CSV measured at a constant retraction speed of 5 μm/s. Wild strain—gray dots; Brazil—yellow dots; South Africa—blue dots; United Kingdom—purple dots. (E) Mean +/– SE of rupture forces measured between all S1 RBD proteins and CSV. (F) Box plot of specific binding probabilities between the wild-strain S1 RBD and wild-type (mEF +/+) and Vim null (mEF −/−) cells. Force maps were collected on top of confluent mEF +/+ and mEF −/– at two different retraction probe speeds—1 and 5 μm/s. One data point in each box denotes the percentage of all specific adhesive force curves in relation to all force curves collected. By using cell lines that lack ACE2 expression, we could eliminate confounding ACE2 interference in our measurements. An additional control experiment using cells without Vim expression gave us confidence that the identified specific interactions can be assigned to S1 RBD-CSV. The significance of differences was determined using the two-tailed Student’s *t*-test, where *p* ≤ 0.05 was considered to be statistically significant.

Figure 3A shows immunostaining of extracellular Vim expressed in wild-type mEF cells (mEF +/+) and the intracellular vimentin cytoskeleton in the same cell type. Panel (3A1) shows predominant perinuclear localization of extracellular Vim on the surface of non-permeabilized cells subjected to vital staining. Extracellular Vim is not evenly distributed over the entire cell surface, which is in good agreement with Vim distribution reported in previous work,^11^ in which cell-surface vimentin (CSV) on human embryonic kidney epithelial and lung cancer cells was detected and visualized.

Permeabilized cells with Vim fibers surrounding the nuclei (blue), extending to the cell periphery, are shown in panel (A2). mEF +/+ cells incubated only with a secondary antibody are presented in panel (A3), thus serving as a control for Figure 3A1. The lack of Vim expression by Vim null cells (mEF −/−) was also confirmed in panel (A4), which shows mouse embryonic fibroblasts without Vim expression treated with both anti-Vim primary and secondary antibodies. The same AFM experimental setup used for Vim-covered mica surfaces was used with live cells (mEF +/+ and mEF −/−) (Figure 3B). Figure 3C shows the distribution of rupture forces between different variants of S1 RBD and Vim-expressing mEF cells (mEF +/+) as a function of natural logarithm of loading rates. Every point on the figure corresponds to a single force–distance curve. Data points for three different S1 RBD VOC (Brazil—yellow, South Africa—blue, and U.K.—purple dots) are gathered toward the center of the plot because they were collected using a single approach speed of 5 μm/s, whereas the wild-type strain S1 RBD was measured at 1, 5, and 10 μm/s /retraction speeds. In live cell measurements, the calculated energy activation barrier width was 0.4 ± 0.03 nm for S1 RBD and CSV, and the off-rate constant *k*_off_ (dissociation rate) was 0.19 ± 0.07 s^–1^.

Figure 3D presents rupture forces for wild-type strain and three VOC S1 RBD binding to extracellular Vim expressed on the surface of mEF +/+ cells (all data points) measured for a single retraction speed of 5 μm/s. With both purified protein systems and live Vim-expressing cells, our study revealed specific and strong interactions between the S1 RBD and Vim (Figures 1–3), characterized by similar patterns of molecular interactions between proteins immobilized on the mica surface and expressed in living cells. The biophysical characteristics of the bonds were similar in both models—mica and cellular surface. The fact that the S1 RBD–Vim bond strength is proportional to the logarithm of the loading rate confirms the application of the Bell–Evans model.^17,23,26^ Similar molecular interactions were also reported between the S1 protein and ACE2 in artificial surfaces and live cells^4^ and in other studies focused on the virus–receptor interaction.^27,28^ Interestingly, the calculated distance to the transition state is smaller for S1 RBD/Vim interactions compared to S1 RBD/ACE2 interactions,^4^ indicating that a narrower energy valley describes the energy landscape. This suggests that the S1 RBD and Vim interactions lead to more rigid and compact bonds.^27^

According to our results, the unbinding force between S1 RBD and Vim is lower when measured using the cellular model (CSV) than the isolated molecular model with Vim immobilized on top of the mica surface. The possible source of this discrepancy may be differences in presentation of extracellular Vim on top of the cells, which, unlike the model in which Vim is immobilized on the mica surface, allows for deformations of the Vim oligomers and the cell surface when binding/unbinding occurs. Local cell membrane deformations are able to dissipate the applied stress during AFM tip pushing and reveal the mechanical resilience of the protein complex during AFM tip pulling backward. This can be confirmed by the greater distance to the transition state (to the unbound state) and the longer binding time observed for S1 RBD and Vim interactions in the cellular model compared to the isolated protein model.

**Figure 4:**
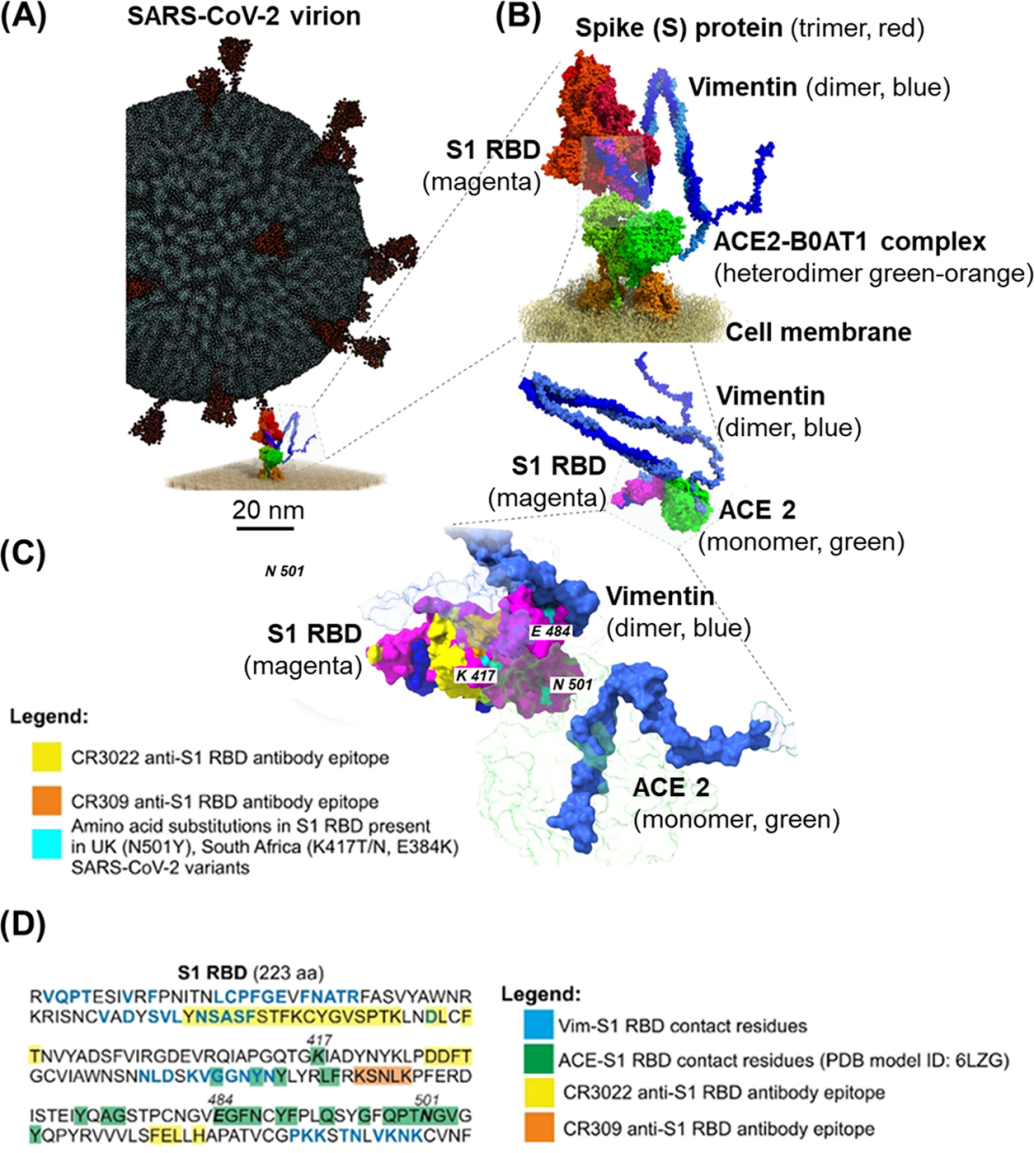
Visualization of putative interaction scenario between the SARS-CoV-2 virion–ACE2 receptor complexed with the B0AT1 amino acid transporter anchored in the cell membrane and Vim dimer (A, B). The model was inferred from the Vim dimer-S1 RBD docking prediction model (C), and alignment of the obtained complex to the experimentally determined structures of (i) ACE2-S1 RBD (PDB model ID: 6LZG), (ii) SARS-CoV-2 S-ACE2 (PDB model ID: 7DF4), (iii) ACE2-B0AT1 heterodimer (PDB model ID: 6M18) inserted into cell membrane model derived from the MemProtMD database,^31^ and (iv) a coarse grain model of the SARS-CoV-2 virion.^32^ 3D structures of the Vim dimer (466 aa) and S1 RBD (223 aa) were predicted using AlphaFold v2.2.2 tool.^33^ The docking between the C-terminal part of Vim (390–466 aa) and S1 RBD was performed using HADDOCK v2.4 software with default parameters^34^ on the basis of the active protein residues, i.e., those creating contacts between the proteins, identified with CASTp 3.0 tool.^35^ The predicted contact residues between Vim-S1 RBD and ACE2-S1 RBD (PDB model ID: 6LZG), amino acid substitutions present in U.K., South Africa, and Brazil variants of SARS-CoV-2, as well as the epitopes of CR3032 and CR309 anti-S1 RBD antibodies are shown (C, D). PyMOL v2.5.2 software was used to align the protein complexes, prior to repair (--command = RepairPDB) their structures with FoldX v5.^36^ ChimeraX v.1.4^37^ was used to visualize the aligned models.

As shown in Figure 3E, the mean values of the rupture forces between all S1 RBD variants and CSV are similar and range from 25.8 pN for the South African variant to 31.3 pN for the Brazilian variant. This similarity indicates that the relevant mutations, i.e., K417N/T, E484K, and N501Y, have no significant impact on Vim-binding affinity and capacity. In the case of ACE2 binding, K417N/T and E484K have little effect on affinity or even decrease it (K417N).^29,30^ However, the N501Y mutation enhances the affinity of S-protein for ACE2 by 7-fold.^30^ The K417N/T, E484K, and N501Y mutations involve residues that directly interact with ACE2 (Figure 4C,D), but according to our in silico S1 RBD–Vim docking model, none of these mutated residues is in contact with Vim (Figure 4C,D) since ACE2 and Vim binding interfaces are localized on the opposite sites of S1 RBD (Figure 4).

Analysis of SARS-CoV-2 S1 RBD and CSV interactions allowed us to precisely sort out the adhesion forces in the cellular model. Figure 3F shows the specific binding probabilities (BP) measured by the S1 RBD-functionalized AFM probe and mEF cells with (+/+) or without (−/−) Vim expression. Specific adhesion events for mEF +/+ cells were observed on up to 10% of the force curves for retraction speed at 1 and 5 μm/s. The binding frequency observed during the S1 RBD and mEF −/– cell interactions (control experiments) is significantly lower with specific adhesion events for mEF −/– cells observed only on 0–4% of the total force curves. The difference in probability of S1 RBD–Vim binding for cells with and without Vim expression confirms specific binding between S1 RBD and CSV. In our experiments, the binding probability is typical for AFM single-molecule experiments and reaches 10%.^4,27^

### 3.3 Computer Modeling

In addition, computer modeling of potential S1 RBD docking sites with a Vim dimer was performed (Figure 4). The focus was on the potential binding of S1 to the C-terminal portion of Vim. The results of our previous studies^11^ suggest that antibodies targeting the C-terminal tail domain of Vim are most effective in blocking SARS-CoV-2 host cell invasion. Computer 3D analysis indicates active residues in both proteins, i.e., those creating contacts between the proteins. We found pseudobonds (highlighted in yellow in Figure 4) between S1 RBD and Vim. Although it is a theoretical docking model, the recent results by Amraei et al.^12^ indicating extracellular Vim as a SARS-CoV-2 attachment factor provide indirect evidence of its plausibility. Briefly, the authors showed that the S1 RBD is sufficient for the S-protein interaction with Vim, but more importantly, that CR3022 anti-S1 RBD antibody (but not CR309) does not interfere with S1 RBD binding to ACE2; however, it inhibits its interaction with Vim. The former observation indicates that S1 RBD recognizes ACE2 and Vim by different binding interfaces (paratopes). Indeed, in our S1 RBD–Vim docking model, only S1 RBD residues recognized by CR3022 are in contact with Vim (Figure 4C,D). In fact, they are part of the largest (18 aa) epitope recognized by CR3022, i.e., Y***NSASF***STFKCYGVSPTK (bolded and italicized residues are part of the Vim binding interface).

## 4 Conclusions

In conclusion, our studies of the biomechanics and kinetics of the interactions between the S1 RBD of wild-type and selected mutants of SARS-CoV-2 and Vim, using AFM and QCM-D methods, have provided new evidence for a mechanically strong and specific binding of the S1 RBD to Vim, which confirms the results of previous biological studies that identified Vim as a possible attachment side of viral entry into host cells. Moreover, the putative interaction scenario between SARS-Cov-2–ACE2 receptor and Vim dimer was explored in situ, verifying possible contact residues between Vim-S1 RBD and ACE2-S1 RBD. The results of our study will help to fully understand the mechanisms of virus entry into host cells and to develop safe and effective methods of prevention, as well as new methods of treatment of Covid-19. Study of interface interaction between Vim and viral fusion proteins can be also used to develop new functional antiviral materials or production of nanoparticles that will mimic the virion of the SARS-CoV-2 virus and might be used in biological research in laboratories without access to Biological Safety Level 3 that is required for working with native SARS-CoV-2 strains, which is particularly important given the fact that diseases caused by the SARS-CoV-2 virus and subsequent mutations will continue to be one of the major challenges facing medicine in the coming years.

## Supporting Information Available

Additional experimental results—interactions between the S1 RBD and ACE2 (PDF)

## References

[ref1] Masre S. F.; Jufri N. F.; Ibrahim F. W.; Raub S. H. A. Classical and Alternative Receptors for SARS-CoV-2 Therapeutic Strategy. Rev. Med. Virol. 2021, 31, 1–9. 10.1002/rmv.2207. PMC7883063 33368788

[ref2] Jackson C. B.; Farzan M.; Chen B.; Choe H. Mechanisms of SARS-CoV-2 Entry Into Cells. Nat. Rev. Mol. Cell Biol. 2022, 23, 3–20. 10.1038/s41580-021-00418-x. 34611326 PMC8491763

[ref3] Shang J.; Wan Y.; Luo C.; Ye G.; Geng Q.; Auerbach A.; Li F. Cell Entry Mechanisms of SARS-CoV-2. Proc. Natl. Acad. Sci. U.S.A. 2020, 117, 11727–11734. 10.1073/pnas.2003138117. 32376634 PMC7260975

[ref4] Yang J.; Petitjean S. J.; Koehler M.; Zhang Q.; Dumitru A. C.; Chen W.; Derclaye S.; Vincent S. P.; Soumillion P.; Alsteens D. Molecular Interaction and Inhibition of SARS-CoV-2 Binding to the ACE2 Receptor. Nat. Commun. 2020, 11, 4541 10.1038/s41467-020-18319-6. 32917884 PMC7486399

[ref5] Cuervo N. Z.; Grandvaux N. ACE2: Evidence of Role as Entry Receptor for SARS-CoV-2 and Implications in Comorbidities. eLife 2020, 9, e61390 10.7554/eLife.61390. 33164751 PMC7652413

[ref6] Yu Y. T.-C.; Chien S.-C.; Chen I.-Y.; Lai C.-T.; Tsay Y.-G.; Chang S. C.; Chang M.-F. Surface Vimentin is Critical for the Cell Entry of SARS-CoV. J. Biomed. Sci. 2016, 23, 14 10.1186/s12929-016-0234-7. 26801988 PMC4724099

[ref7] Zhang Y.; Wen Z.; Shi X.; Liu Y.-J.; Eriksson J. E.; Jiu Y. The Diverse Roles and Dynamic Rearrangement of Vimentin During Viral Infection. J. Cell Sci. 2021, 134, jcs250597 10.1242/jcs.250597. 33154171

[ref8] Ramos I.; Stamatakis K.; Oeste C. L.; Pérez-Sala D. Vimentin as a Multifaceted Player and Potential Therapeutic Target in Viral Infections. Int. J. Mol. Sci. 2020, 21, 4675 10.3390/ijms21134675. 32630064 PMC7370124

[ref9] Du N.; Cong H.; Tian H.; Zhang H.; Zhang W.; Song L.; Tien P. Cell Surface Vimentin is an Attachment Receptor for Enterovirus 71. J. Virol. 2014, 88, 5816–5833. 10.1128/JVI.03826-13. 24623428 PMC4019121

[ref10] Thalla D. G.; Jung P.; Bischoff M.; Lautenschläger F. Role of Extracellular Vimentin in Cancer-Cell Functionality and its Influence on Cell Monolayer Permeability Changes Induced by SARS-CoV-2 Receptor Binding Domain. Int. J. Mol. Sci. 2021, 22, 7469 10.3390/ijms22147469. 34299089 PMC8303762

[ref11] Suprewicz Ł.; Swoger M.; Gupta S.; Piktel E.; Byfield F. J.; Iwamoto D. V.; Germann D.; Reszeć J.; Marcińczyk N.; Carroll R. J.; et al. Extracellular Vimentin as a Target Against SARS-CoV-2 Host Cell Invasion. Small 2022, 18, 2105640 10.1002/smll.202105640. PMC9252327 34866333

[ref12] Amraei R.; Xia C.; Olejnik J.; White M. R.; Napoleon M. A.; Lotfollahzadeh S.; Hauser B. M.; Schmidt A. G.; Chitalia V.; Mühlberger E.; et al. Extracellular Vimentin is an Attachment Factor That Facilitates SARS-CoV-2 Entry Into Human Endothelial Cells. Proc. Natl. Acad. Sci. U.S.A. 2022, 119, e2113874119 10.1073/pnas.2113874119. 35078919 PMC8833221

[ref13] Delguste M.; Peerboom N.; Le Brun G.; Trybala E.; Olofsson S.; Bergström T.; Alsteens D.; Bally M. Regulatory Mechanisms of the Mucin-Like Region on Herpes Simplex Virus During Cellular Attachment. ACS Chem. Biol. 2019, 14, 534–542. 10.1021/acschembio.9b00064. 30735356

[ref14] Lan J.; Ge J.; Yu J.; Shan S.; Zhou H.; Fan S.; Zhang Q.; Shi X.; Wang Q.; Zhang L.; Wang X. Structure of the SARS-CoV-2 Spike Receptor-Binding Domain Bound to the ACE2 Receptor. Nature 2020, 581, 215–220. 10.1038/s41586-020-2180-5. 32225176

[ref15] Wildling L.; Unterauer B.; Zhu R.; Rupprecht A.; Haselgrübler T.; Rankl C.; Ebner A.; Vater D.; Pollheimer P.; Pohl E. E.; et al. Linking of Sensor Molecules With Amino Groups to Amino-Functionalized AFM Tips. Bioconjugate Chem. 2011, 22, 1239–1248. 10.1021/bc200099t. PMC3115690 21542606

[ref16] Kubiak K.; Adamczyk Z.; Oćwieja M. Kinetics of Silver Nanoparticle Deposition at PAH Monolayers: Reference QCM Results. Langmuir 2015, 31, 2988–2996. 10.1021/la504975z. 25692665

[ref17] Senkara-Barwijuk E.; Kobiela T.; Lebed K.; Lekka M. Reaction Pathway and Free Energy Profile Determined for Specific Recognition of Oligosaccharide Moiety of Carboxypeptidase Y. Biosens. Bioelectron. 2012, 36, 103–109. 10.1016/j.bios.2012.04.014. 22541811

[ref18] Wasilewska M.; Żeliszewska P.; Pogoda K.; Deptuła P.; Bucki R.; Adamczyk Z. Human Vimentin Layers on Solid Substrates: Adsorption Kinetics and Corona Formation Investigations. Biomacromolecules 2022, 23, 3308–3317. 10.1021/acs.biomac.2c00415. 35829774 PMC9364323

[ref19] Adamczyk Z. Protein Adsorption: A Quest for a Universal Mechanism. Curr. Opin. Colloid Interface Sci. 2019, 41, 50–65. 10.1016/j.cocis.2018.11.004.

[ref20] Bratek-Skicki A.; Sadowska M.; Maciejewska-Prończuk J.; Adamczyk Z. Nanoparticle and Bioparticle Deposition Kinetics: Quartz Microbalance Measurements. Nanomaterials 2021, 11, 145 10.3390/nano11010145. 33435619 PMC7827609

[ref21] Kubiak K.; Adamczyk Z.; Cieśla M. Fibrinogen Adsorption Mechanisms at the Gold Substrate Revealed by QCM-D Measurements and RSA Modeling. Colloids Surf., B 2016, 139, 123–131. 10.1016/j.colsurfb.2015.11.052. 26705826

[ref22] Kubiak K.; Adamczyk Z.; Maciejewska J.; Oćwieja M. Gold Nanoparticle Monolayers of Controlled Coverage and Structure. J. Phys. Chem. C 2016, 120, 11807–11819. 10.1021/acs.jpcc.6b02683.

[ref23] Hane F. T.; Attwood S. J.; Leonenko Z. Comparison of Three Competing Dynamic Force Spectroscopy Models to Study Binding Forces of Amyloid-β (1–42). Soft Matter 2014, 10, 1924–1930. 10.1039/c3sm52257a. 24652035

[ref24] Bell G. I. Models for the Specific Adhesion of Cells to Cells. Science 1978, 200, 618–627. 10.1126/science.347575. 347575

[ref25] Evans E.; Ritchie K.; Merkel R. Sensitive Force Technique to Probe Molecular Adhesion and Structural Linkages at Biological Interfaces. Biophys. J. 1995, 68, 2580–2587. 10.1016/S0006-3495(95)80441-8. 7647261 PMC1282168

[ref26] Bullerjahn J. T.; Sturm S.; Kroy K. Theory of Rapid Force Spectroscopy. Nat. Commun. 2014, 5, 4463 10.1038/ncomms5463. 25079911 PMC4124868

[ref27] Koehler M.; Aravamudhan P.; Guzman-Cardozo C.; Dumitru A. C.; Yang J.; Gargiulo S.; Soumillion P.; Dermody T. S.; Alsteens D. Glycan-Mediated Enhancement of Reovirus Receptor Binding. Nat. Commun. 2019, 10, 4460 10.1038/s41467-019-12411-2. 31575869 PMC6773860

[ref28] Delguste M.; Zeippen C.; Machiels B.; Mast J.; Gillet L.; Alsteens D. Multivalent Binding of Herpesvirus to Living Cells is Tightly Regulated During Infection. Sci. Adv. 2018, 4, eaat1273 10.1126/sciadv.aat1273. 30128355 PMC6097811

[ref29] Gan H. H.; Twaddle A.; Marchand B.; Gunsalus K. C. Structural Modeling of the SARS-CoV-2 Spike/Human ACE2 Complex Interface Can Identify High-Affinity Variants Associated with Increased Transmissibility. J. Mol. Biol. 2021, 433, 167051 10.1016/j.jmb.2021.167051. 33992693 PMC8118711

[ref30] Laffeber C.; de Koning K.; Kanaar R.; Lebbink J. H. Experimental Evidence for Enhanced Receptor Binding by Rapidly Spreading SARS-CoV-2 Variants. J. Mol. Biol. 2021, 433, 167058 10.1016/j.jmb.2021.167058. 34023401 PMC8139174

[ref31] Stansfeld P. J.; Goose J. E.; Caffrey M.; Carpenter E. P.; Parker J. L.; Newstead S.; Sansom M. S. P. MemProtMD: Automated Insertion of Membrane Protein Structures into Explicit Lipid Membranes. Structure 2015, 23, 1350–1361. 10.1016/j.str.2015.05.006. 26073602 PMC4509712

[ref32] Yu A.; Pak A. J.; He P.; Monje-Galvan V.; Casalino L.; Gaieb Z.; Dommer A. C.; Amaro R. E.; Voth G. A. A Multiscale Coarse-Grained Model of the SARS-CoV-2 Virion. Biophys. J. 2021, 120, 1097–1104. 10.1016/j.bpj.2020.10.048. 33253634 PMC7695975

[ref33] Jumper J.; Evans R.; Pritzel A.; Green T.; Figurnov M.; Ronneberger O.; Tunyasuvunakool K.; Bates R.; Zidek A.; Potapenko A.; Bridgland A.; Meyer C.; Kohl S. A. A.; Ballard A. J.; Cowie A.; Romera-Paredes B.; Nikolov S.; Jain R.; Adler J.; Back T.; Petersen S.; Reiman D.; Clancy E.; Zielinski M.; Steinegger M.; Pacholska M.; Berghammer T.; Bodenstein S.; Silver D.; Vinyals O.; Senior A. W.; Kavukcuoglu K.; Kohli P.; Hassabis D. Highly Accurate Protein Structure Prediction with AlphaFold. Nature 2021, 596, 583–589. 10.1038/s41586-021-03819-2. 34265844 PMC8371605

[ref34] van Zundert G. C. P.; Rodrigues J.; Trellet M.; Schmitz C.; Kastritis P. L.; Karaca E.; Melquiond A. S. J.; van Dijk M.; de Vries S. J.; Bonvin A. The HADDOCK2.2 Web Server: User-Friendly Integrative Modeling of Biomolecular Complexes. J. Mol. Biol. 2016, 428, 720–725. 10.1016/j.jmb.2015.09.014. 26410586

[ref35] Tian W.; Chen C.; Lei X.; Zhao J.; Liang J. CASTp 3.0: Computed Atlas of Surface Topography of Proteins. Nucleic Acids Res. 2018, 46, W363–W367. 10.1093/nar/gky473. 29860391 PMC6031066

[ref36] Schymkowitz J.; Borg J.; Stricher F.; Nys R.; Rousseau F.; Serrano L. The FoldX Web Server: an Online Force Field. Nucleic Acids Res. 2005, 33, W382–W388. 10.1093/nar/gki387. 15980494 PMC1160148

[ref37] Pettersen E. F.; Goddard T. D.; Huang C. C.; Couch G. S.; Greenblatt D. M.; Meng E. C.; Ferrin T. E. UCSF Chimera--a Visualization System for Exploratory Research and Analysis. J. Comput. Chem. 2004, 25, 1605–1612. 10.1002/jcc.20084. 15264254

