# Supplementary material for: Physicochemical Nature of SARS-CoV-2 Spike Protein Binding to Human Vimentin
Source: ACS Appl Mater Interfaces. 2023 Jul 6;15(28):34172–80. doi: 10.1021/acsami.3c03347 (PMC10360031; doi:10.1021/acsami.3c03347)
Supplement: Supplementary file 1 — am3c03347_si_001.pdf [file am3c03347_si_001.pdf]

Physicochemical nature of SARS-CoV-2 spike protein binding to human vimentin

Piotr Deptuła [a], Krzysztof Fiedoruk [b], Monika Wasilewska [c], Łukasz Suprewicz [b], Mateusz Cieśluk [b], Paulina Żeliszewska [c], Magdalena Oćwieja [c], Zbigniew Adamczyk [c], Katarzyna Pogoda [d], Robert Bucki [b*]*

a) Independent Laboratory of Nanomedicine, Medical University of Bialystok, PL-15222 Białystok, Poland

b) Department of Medical Microbiology and Nanobiomedical Engineering, Medical University of Bialystok, PL-15222 Białystok, Poland

c) J. Haber Institute of Catalysis and Surface Chemistry Polish Academy of Science, Niezapominajek 8, PL-30239 Krakow, Poland

d) Institute of Nuclear Physics Polish Academy of Sciences, PL-31342 Krakow, Poland

Corresponding Author

Robert Bucki, Department of Microbiological and Nanobiomedical Engineering, Medical University of Bialystok, Mickiewicza 2c, 15-222 Bialystok, Poland. Phone: (48) 85 748 54 83; email: buckirobert@gmail.com, Katarzyna Pogoda, Institute of Nuclear Physics Polish Academy of Sciences, PL-31342 Krakow, Poland. email: katarzyna.pogoda@ifj.edu.pl

Supporting Information:

Interactions between S1 RBD and ACE2

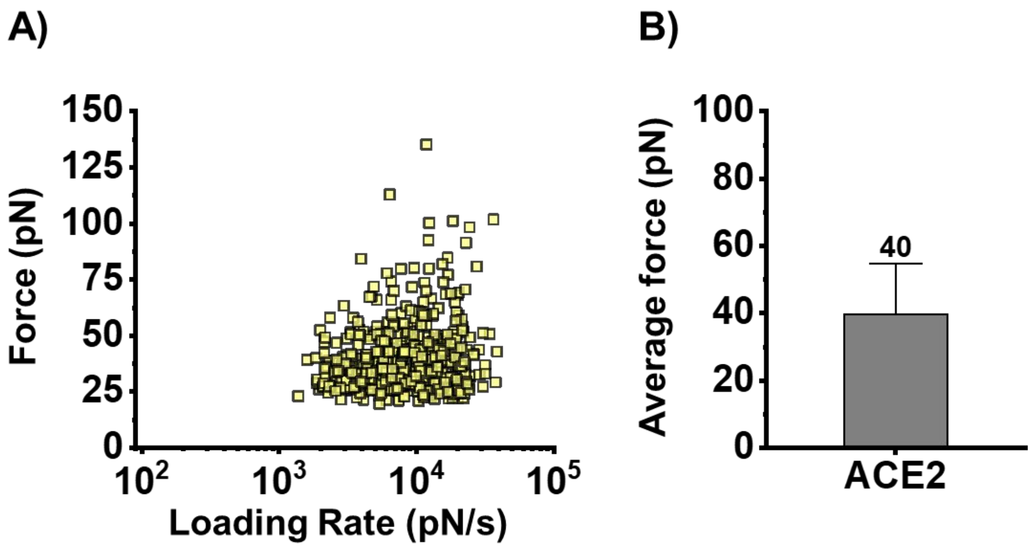

Figure S1. Interactions between S1 RBD and ACE2 measured using the AFM method. A) The distribution of the rupture forces as a function of their log LR measured between S1 RBD and ACE2 for the AFM approach/retract speed equal to 5 μm/s. B) Rupture force of S1 RBD and ACE2 interactions.
